# Stability Computationsfor Isomersof La@C*_n_* (*n* = 72, 74, 76) 

**DOI:** 10.3390/molecules171113146

**Published:** 2012-11-05

**Authors:** Zdeněk Slanina, Filip Uhlík, Shyi-Long Lee, Ludwik Adamowicz, Takeshi Akasaka, Shigeru Nagase

**Affiliations:** 1 Life Science Center of Tsukuba Advanced Research Alliance, University of Tsukuba, 1-1-1Tennodai, Tsukuba, Ibaraki 305-8577, Japan; E-Mail: akasaka@tara.tsukuba.ac.jp; 2 Department of Physical and Macromolecular Chemistry, Charles University in Prague, Faculty of Science, Albertov 6, 128 43 Praha 2, Czech Republic; E-Mail: filip.uhlik@natur.cuni.cz; 3 Department of Chemistry and Biochemistry, National Chung-Cheng University, Chia-Yi 62117, Taiwan; E-Mail: chesll@ccu.edu.tw; 4 Department of Chemistry, University of Arizona, Tucson, AZ 85721-0041, USA; E-Mail: ludwik@email.arizona.edu; 5 Fukui Institute for Fundamental Chemistry, Kyoto University, Nishihiraki-cho 34-4, Kyoto 606-8103, Japan; E-Mail: nagase@ims.ac.jp

**Keywords:** metallofullerenes, DFT computations, isomeric stabilities, Gibbs-energy evaluations, IPR and non-IPR fullerene cages

## Abstract

Density-functional theory calculations are presented for low-energy La@C_72_, La@C_74_ and La@C_76_ isomers with IPR (isolated pentagon rule) and non-IPR cages. The relative isomeric production yields at high temperatures are evaluated using the calculated terms, and the relationships to observations are discussed.

## 1. Introduction

There are only two species [1,2] among C_60_–C_96_ empty fullerenes that are not yet isolated—C_72_ and C_74_. However, their metallofullerenes are well known—for example Ca@C_72_ [3], La@C_72_ [4], Ca@C_74_ [5], Sm@C_74_ [6], Sr@C_74_ [7], Ba@C_74_ [8], La@C_74_ [9,10], Eu@C_74_ [11], Yb@C_74_ [12], Sc_2_@C_74_ [13] or Er_3_@C_74_ [14]. Among them, La@C_72_ and La@C_74_ have a special position as their X-ray crystallographic analysis is available [4,10], though for solvent-related derivatives, namely La@C_72_-C_6_H_3_Cl_2_ with a non-IPR (IPR: isolated pentagon rule [15]) carbon cage and La@C_74_-C_6_H_3_Cl_2_ (IPR cage). Finally, while La@C_76_ has also been observed [16,17], its cage structure is not yet known. 

The metallofullerenes based on the C*_n_* (*n* = 72, 74, 76) cages have also been computed [18,19,20,21,22,23,24,25,26]. The first relative isomeric stabilities were evaluated [21,22] for Ca@C_72_ and Ca@C_74_ and it was shown that the non-IPR encapsulations are significant only in Ca@C_72_. The present paper deals with such calculations for La@C_72_, La@C_74_ and La@C_76_, consistently using the Gibbs energies as required by high temperatures [27,28,29,30]. 

## 2. Calculations

There is [17] just one IPR isomer for C_72_ (*D*_6_*_d_*) and C_74_ (*D*_3_*_h_*) while there are two IPR isomers for C_76_ (*D*_2_ and *T_d_* symmetry). In addition, a few relevant (most populated) non-IPR cages are considered here (the cages are labeled by some conventional code numbers or by symmetry groups). 

The geometry optimizations were carried out using density functional theory (DFT), namely employing Becke’s three parameter functional [31] with the non-local Lee–Yang–Parr correlation functional [32] (B3LYP, as we deal with open-shell systems-the unrestricted B3LYP, or UB3LYP, treatment is applied) in the combined basis set of the 3–21G basis for C atoms and a dz basis set for La suggested by Hay and Wadt [33] with the effective core potential (denoted hereby 3–21G~dz). The B3LYP/3–21G~dz geometry optimizations were carried out with the analytically constructed energy gradient as implemented in the Gaussian03 program package [34]. 

In the optimized B3LYP/3–21G~dz geometries, the harmonic vibrational analysis was carried out with the analytical force-constant matrix. In the same B3LYP/3–21G~dz optimized geometries, higher-level single-point energy calculations were also performed, using the standard 6–31G* basis set for Catoms, *i.e*., the B3LYP/6–31G*~dz level. Moreover, the SDD (Stuttgart/Dresden) basis set [35] was also employed (with the SDD effective core potential for La) for comparison. The Gibbs energies were evaluated using the rotational-vibrational partition functions constructed [36] from the calculated structural and vibrational data using the rigid rotator and harmonic oscillator (RRHO) approximation. Although the temperature region where fullerene or metal of ullerene electric-arc synthesis takes place is not yet known, the new observations [37] supply some arguments to expect it to be around or above 1500 K. Thus, the computed results discussed here are also focused on the temperature region. 

Relative concentrations (mole fractions) *x_i_* of *m* isomers can be evaluated [36] through their partition functions *q_i_* and the enthalpies at the absolute zero temperature or ground-state energies ∆H_0,*i*_^o^ (*i.e*., the relative potential energies corrected for the vibrational zero-point energies) by a compact formula:

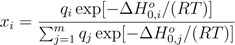
(1)
where *R* is the gas constant and *T* is the absolute temperature. Equation (1) is an exact formula that can be directly derived [36] from the standard Gibbs energies of the isomers, supposing the conditions of the inter-isomeric thermodynamic equilibrium. The electronic partition functions for Equation (1) were constructed by direct summation of the TD B3LYP/3-21G~dz electronic excitation energies. However, the isomeric populations are not very sensitive to the electronic contributions, and therefore the vibrational-rotational partition functions for the electronic excited states are taken as equal to those of the ground state. The chirality contribution was included accordingly [38]. 

Let us note that in addition to the conventional RRHO treatment in Equation (1), a further modified approach for the description of the encapsulate motions can be considered [39], following the findings [40] that the encapsulated atoms can exercise large amplitude motions, especially so at elevated temperatures (unless the motions are restricted by cage derivatizations [41]). One can expect that if the encapsulate is relatively free, then at sufficiently high temperatures its behavior in different cages will bring about the same contribution to the partition functions. However, such uniform contributions would then cancel out in Equation (1). This simplification is called [39] the free, fluctuating, or floating encapsulate model (FEM) and requires two steps. In addition to the removal of the three lowest vibrational frequencies (belonging to the metal motions in the cage), the symmetries of the cages should be treated as the highest (topologically) possible, which reflects the averaging effects of the large amplitude motions. Forexample, for the C_74_ IPR isomer species, the *D*_3_*_h_* symmetry is employed within the FEM scheme, although its statical symmetry (*i.e.*, after the geometry optimization) is just C_2_*_v_*. Generally, the FEM treatment gives a better agreement [42] with the available observed data than the RRHO approach. 

## 3. Results and Discussion

**Figure 1:**
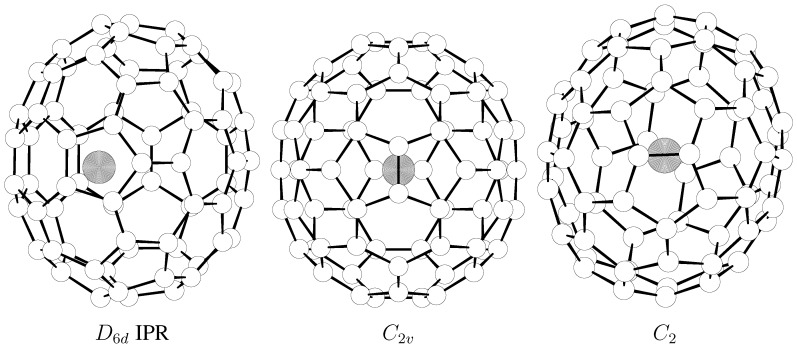
B3LYP/3–21G~dz optimized structures of the three studied La@C_72_ isomers.

**Figure 2:**
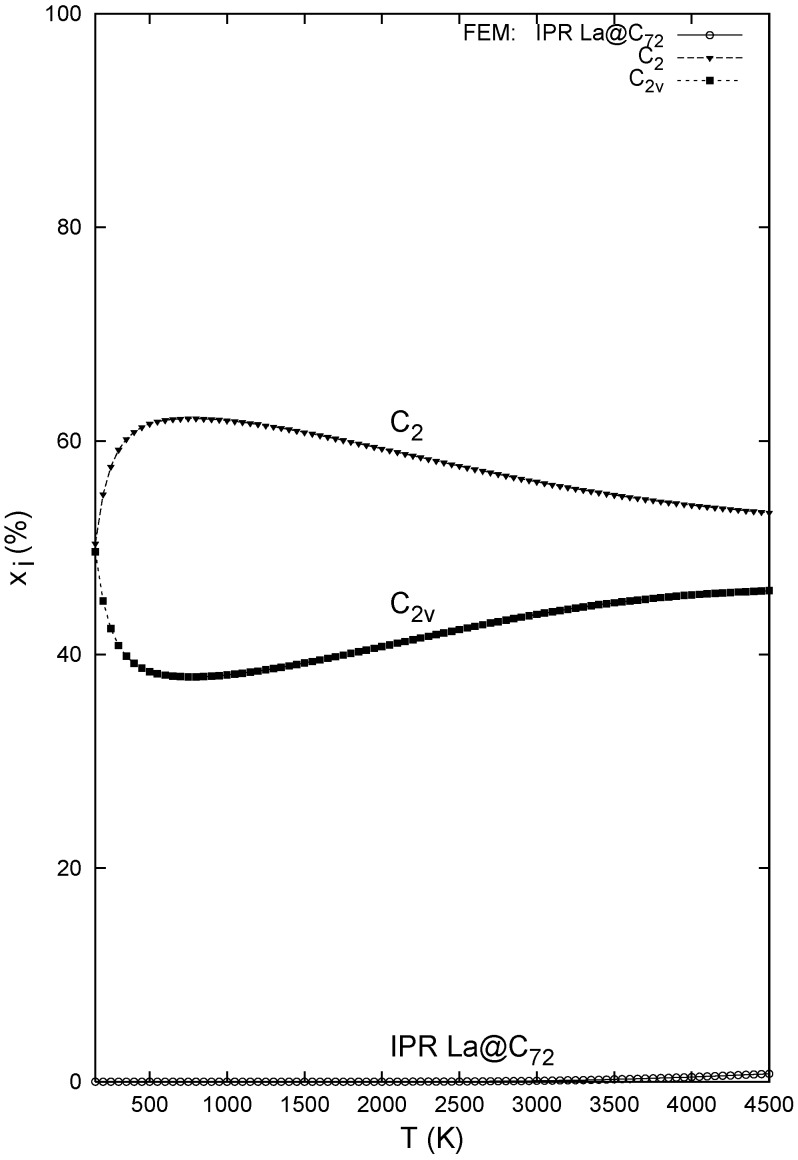
Relative concentrations of the La@C_72_ isomers computed with the B3LYP/6–31G*~dzenergetics, B3LYP/3–21G~dz entropy, and FEM treatment.

Table 1 presents the separation energetics for the three La@C_72_ isomers (Figure 1). As the sole IPR species is considerably higher in energy, it is not significantly populated even at very high temperatures (Figure 2). Although the potential energy difference is very small between the *C*_2_*_v_* and *C*_2_ non-IPR isomers, the *C*_2_-cage-based La@C_72_ structure is always more populated and the *C*_2_ cage is indeed present in the observation [4] (as the only isomer isolated). The effects of the cage derivatizations during chromatographic separation should however also be evaluated as a final step in theory-experiment comparisons. The relative-stability picture is basically the same from the FEM and RRHO treatments as the two decisive non-IPR species do have the same symmetry number in both approaches. The IPR-cage-based La@C_72_ is somewhat more populated in the RRHO approximation owing to symmetry number reduction, but still remains as a very minor isomer. 

**Table 1:** La@C_72_ relative potential energies ∆*E_pot,rel_* computed ^a^ in the B3LYP/3–21G~dz optimized geometries.

Species ^b^	∆ *E_pot,rel_*	(kcal/mol)
	B3LYP/3–21G~dz	B3LYP/6–31G*~dz
IPR	36.80	37.40
C_2_ *_v_*	4.40	0.26
C_2_	0	0

^a^ See the text for the abbreviations; ^b^ See Figure 1.

**Figure 3:**
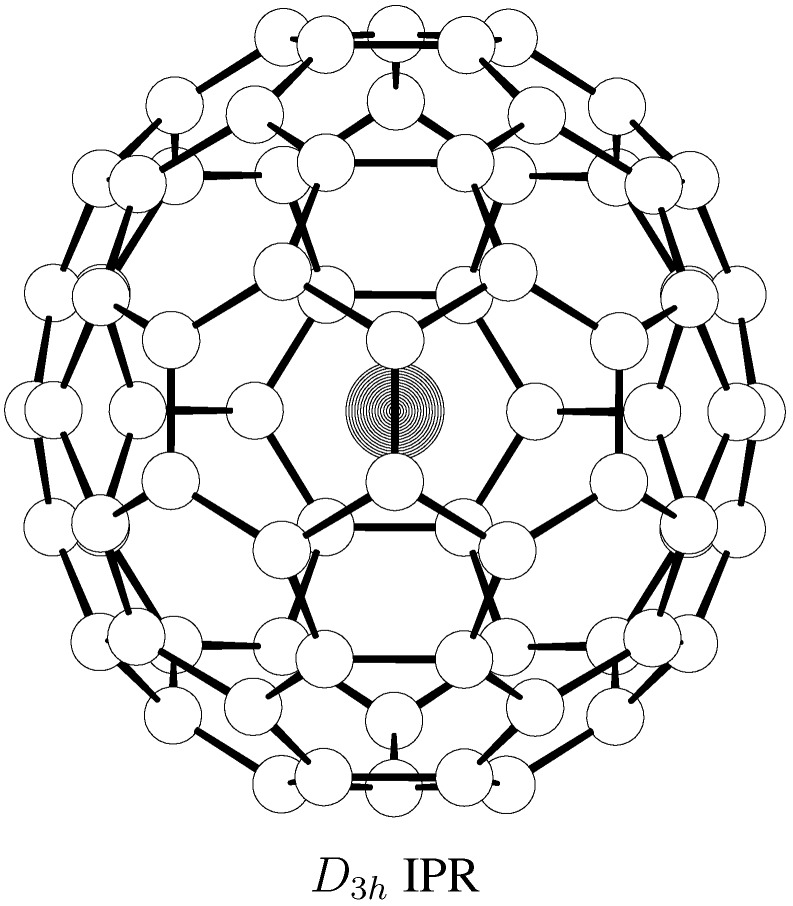
B3LYP/3-21G~dz optimized structure of the observed La@C_74_ IPR-based isomer.

**Figure 4:**
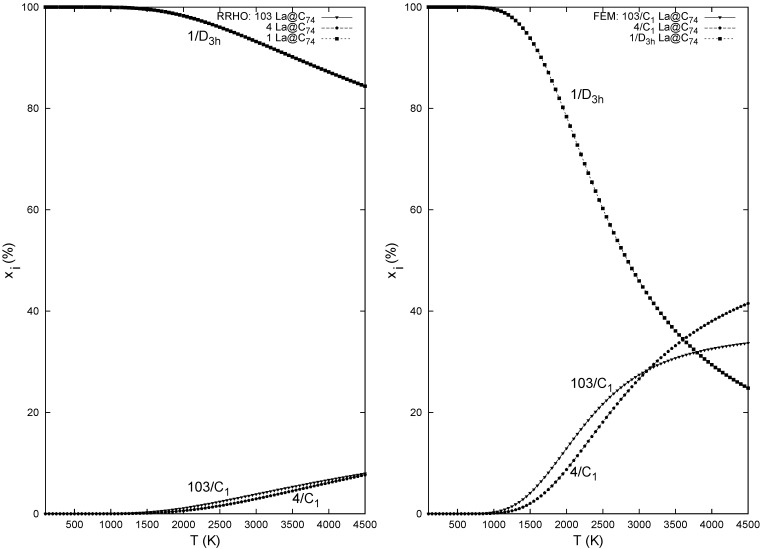
Relative concentrations of the La@C_74_ isomers computed with the B3LYP/6–311G*~sddenergetics, B3LYP/3-21G~dz entropy, and RRHO (left) or FEM (right) treatment.

The role of the sole IPR cage is however quite different in the La@C_74_ isomeric system. The La@C_74_ isomer with the IPR cage (Figure 3) is the lowest in energy [25] and the non-IPR species are located rather high. Temperature developments of the relative concentrations of the three La@C_74_ isomers in the RRHO and FEM treatments are presented in Figure 4—it is a system where the populations from the two approaches are considerably different. In the temperature region around 1500 K, the *D*_3_*_h_* species is clearly prevailing while two non-IPR cage related isomers 103 and 4 represent just a few percent of the equilibrium isomeric mixture. The pronounced difference between the RRHO and FEM treatments is substantially related to the considerable change in the symmetry number of the IPR-cage-based endohedral (from 2 in the rigid RRHO to 6 in the flexible FEM approach). Still, both evaluations are in agreement with the observation [10] in which just one La@C_74_ species was isolated and evidenced by X-ray analysis to contain the IPR carbon cage. According to the calculations, the La endohedrals with non-IPR C_74_ cages could possibly be isolated only with a considerable separation effort (though the derivatization effects by solvent are yet to be evaluated).

**Figure 5:**
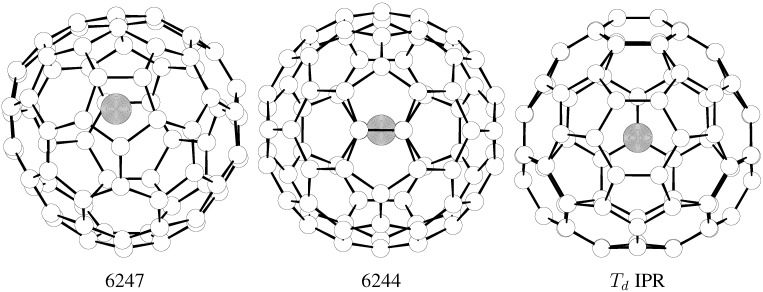
B3LYP/3-21G~dz optimized structures of the three most populated La@C_76_ isomers.

**Figure 6:**
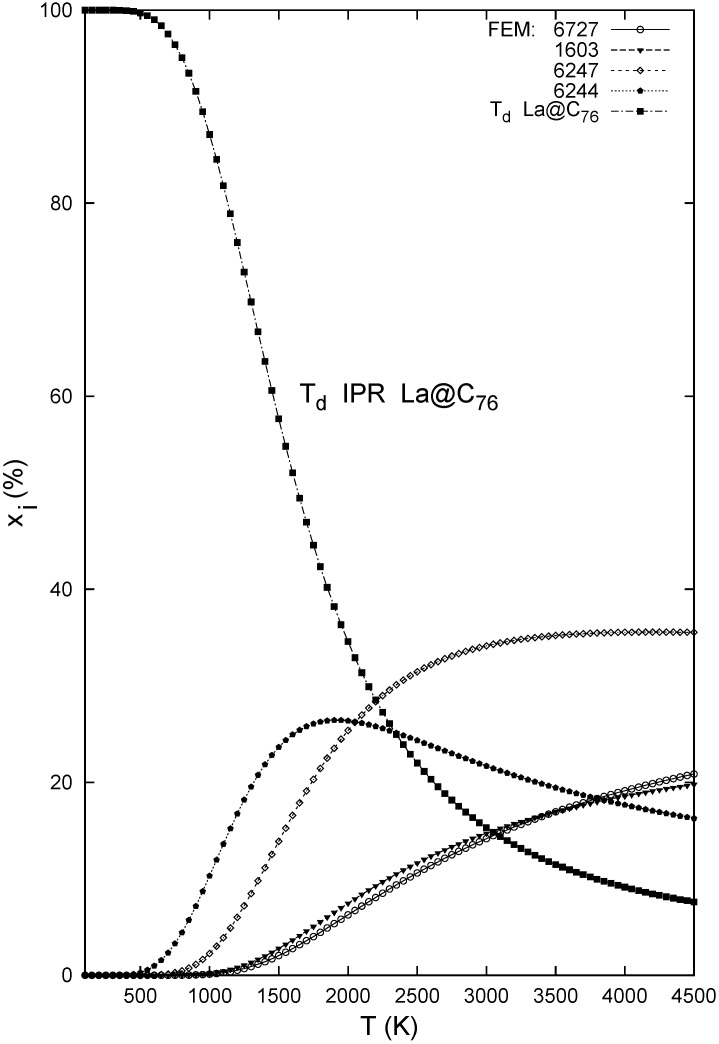
Relative concentrations of the La@C_76_ isomers computed with the B3LYP/6-31G*~dzenergetics, B3LYP/3-21G~dz entropy, and FEM treatment.

The sensitivity to the symmetry representation is even larger in the La@C_76_ isomeric set (Figure 5)—owing to the effective *T_d_* symmetry of the lowest energy species (Table 2) in the FEM approach. Interestingly enough, La encapsulation into the other IPR cage (*D*_2_) produces an endohedral higher by some 20 kcal/mol so that it cannot compete with structure 6244 and a few other non-IPR La@C_76_ isomers (Table 2). Figure 6 demonstrates that while the *T_d_*-IPR-based endohedral is the major isomer in the relevant temperature region, there should be a significant minor isomer, namely derived from the 6244 cage, and possibly even a second minor species related to the 6247 non-IPR cage. However, this computational prediction remains to be checked by observations. Thus, in the studied triad of isomeric systems, La@C_76_ comes rather in the middle, between La@C_72_ as non-IPR controlled and La@C_74_ just the opposite. 

**Table 2:** La@C_76_ relative potential energies ∆*E_pot,rel_* computed ^a^ in the B3LYP/3–21G~dz optimized geometries.

Species ^b^	∆ *E_pot,rel_*	(kcal/mol)
	B3LYP/3–21G *~*dz	B3LYP/6–31G*~dz *^c^*
6727	15.86	19.46
1603	12.26	17.76
6247	10.00	13.22
6244	6.90	7.36
T *_d_* IPR	0	0

^a^ See the text for the abbreviations; ^b^ See Figure 5; ^c^ In the B3LYP/6-31G*~dz optimized geometry.

Various endohedral cage compounds have been suggested as possible candidate species for molecular memories and other future nanotechnological applications. One approach is built on endohedral species with two possible location sites of the encapsulated atom [43] while another concept of quantum computing aims at a usage of spin states [44] or fullerene-based molecular transistors [45]. For the first memory concept, the otherwise low potential barriers for a three-dimensional rotational motion of encapsulates in the cages [46,47] could be restricted by an additional cage derivatization [41]. Obviously, a further accumulation of knowledge on endohedral properties is needed, both computed [48,49,50,51,52,53] and observed [54,55,56], before such applications can be truly explored. 

## 4. Conclusions

The computational study shows that while La@C_72_ is dominated by non-IPR cages and in La@C_74_ the IPR moiety prevails, for La@C_76_ a non-IPR cage should be present as a minor isomer. The sensitivity of the computed populations to the symmetry treatment is also highlighted. 
